# Lipid levels in the Jiarong Tibetan’s diet at high altitudes: a cross-sectional survey

**DOI:** 10.3389/fnut.2023.1207710

**Published:** 2023-06-26

**Authors:** Tang Xiaoyue, Qiao Qichuan, Guo Jing, Sanlang Pengcuo, Huang Yu, Li Tingxin

**Affiliations:** ^1^Department of Health Management and Physical Examination, Sichuan Provincial People’s Hospital, University of Electronic Science and Technology of China, Chengdu, China; ^2^Department of Clinical Nutrition, Yizheng Hospital of Nanjing Drum Tower Hospital Group, Yizheng, Jiangsu, China; ^3^Chinese Academy of Sciences Sichuan Translational Medicine Research Hospital, Chengdu, China; ^4^Physical Examination, Aba Prefecture People’s Hospital, Maerkang, China

**Keywords:** lipid metabolism, unsaturated fatty acid, high-fat diet, Tibetan, altitude

## Abstract

Despite the ongoing debate on the inconsistent and controversial effects of Tibetan diet on blood lipid levels at high altitude, this cross-sectional study was conducted to analyze the relationship between dietary practices and blood lipid levels among Jiarong Tibetan population. A total of 476 Jiarong Tibetan residents were included, in which basic demographic data, physical activity records, simplified food frequency questionnaire, and biochemical data were collected. Using multivariate logistic regression analysis, the potential associations between the variables were examined, and it was found that fat energy supply ratio increased with the elevation of altitude, while the lipid level showed an inverted U-shaped variation. However, the findings suggested that a diet rich in unsaturated fatty acids might balance the effects of the Tibetan diet on the risk of lipid metabolism disorders. Therefore, it is crucial to concentrate on the fat composition rather than the amount of fat E% intake on the plateau. The results highlighted the importance of investigating the interaction between environment and genes in lipid levels among plateau Tibetan population. However, further large-scale prospective studies are required for better understanding of the complexities involved in dietary practices and their influences on blood lipid levels.

## 1. Introduction

According to the available data from a reliable source (1), it was estimated that about 7% of the world’s population, equivalent to approximately 500.3 million people, live in areas located at an altitude of 1,500 meters above sea level. Furthermore, the data suggested that there are roughly 81.6 million individuals living at elevations higher than 2,500 meters and more than 14.4 million people residing at heights exceeding 3,500 meters (2). Several studies have shown that high-altitude environments may have some effects on the human health (3–6). However, the conclusions of effects of Tibetan diet on blood lipid levels in high altitude are inconsistent and controversial (7–10).

Tibetans constitute one of the earliest ethnic communities in China and South Asia, with the majority residing in high-altitude regions. Their traditional dietary habits primarily revolve around dairy products and meat consumption (11). The incidence of cardiovascular diseases (CVDs) and metabolic syndrome within the Tibetan population is comparable to that observed in other ethnic groups (10, 12). Previous research has mentioned the features of physiological markers, genetic factors, and gut microorganisms among Tibetans, which are deemed to be adaptive responses to their surroundings (13). However, in the majority of cases, the data were not compared with individuals of the same race, such as those belonging to immigrant populations (14, 15), the Han (16, 17) or Mongolian populations (18). To comprehensively analyze the effects of Tibetan diet, which is distinctive due to its strong local characteristics, on changes in physiological indicators, it is essential to take into account various factors such as ethnic differences, behavioral habits, and other relevant contextual variables (19). To our knowledge, there is no existing literature that specifically examines the relationship between Tibetan diet and blood lipid levels within genetically homogeneous groups living at various altitudes.

JThe Jiarong Tibetan population is primarily located in Ngawa Tibetan Autonomous Prefecture, which is situated in the Minjiang River basin of southwestern China. This region is adjacent to both the Tibetan Plateau and the Chengdu Plain, with altitudes ranging from 500 to 4,000 meters. The Jiarong Tibetan people are believed to originate from an amalgamation of genetic materials from both Qiang and Tubo populations after Tubo migrated eastward during the Tang Dynasty (20). The Jiarong Tibetan branch has coexisted with the Han and Qiang ethnic groups for a noticeable period. Their distribution area encompasses both plains and mountains, where they were engaged in both nomadic and agricultural practices. This particular Tibetan branch is characterized by its exceptional customs and living habits. The variance in altitude across the habitation range provides a unique opportunity to study adaptive changes within a single ethnic group at different elevations.

The present study aimed to investigate the association of Tibetan diet with lipid level in the Jiarong Tibetan population exposed to different altitudes. By eliminating the influences of ethnic, religious, and traditional cultures, the findings of this study would prove beneficial in analyzing the altitude adaptation of the Jiarong Tibetan population for the purpose of improving the healthcare management.

## 2. Materials and methods

### 2.1. Study participants

From September 2018 to June 2019, the cross-sectional study, based on a multistage stratified clustering sampling, was conducted in the Jiarong Tibetan area. The study sample size, exclusion criteria, and inclusion criteria were previously described (12). The allowable error *δ* was set to 5%, and the minimum sample size was calculated as 384 according to the following formula: $n=0.25\times {\left(\frac{\mu }{\delta }\right)}^{2}$ . To account for the unavoidable loss of survey samples, we ensured to incorporate a minimum of 500 samples. Within the scope of 12 counties, the main settlements of Jiarong Tibetans were included randomly. To account for the unavoidable loss of survey samples, we ensured to incorporate a minimum of 500 samples. Within the scope of 12 counties, the main settlements of Jiarong Tibetans were included randomly. Subjects were mainly from seven high-altitude areas, including Maerkang city, Rangtang county, Jinchuan county, Aba county, Hongyuan county, Xiaojin county, and Maoxian county (Supplementary Figure S1). We categorized these areas into three distinct groups based on their altitude: 1,500 ~ 2,399 m, 2,400 ~ 3,299 m, and 3,300 m and above. These groups were labeled as low-altitude group (LA group), medium-altitude group (MA group), and high-altitude group (HA group), respectively. The average elevation of each county is shown in Supplementary Figure S1, with the red star indicating the sampled counties. After comprehending the objectives and potential benefits of our study, all subjects willingly signed an informed consent form. Therefore, the participants provided their informed consent. The study was approved by the Human Ethics and Research Ethics committees of Sichuan Provincial People’s Hospital (Chengdu, China; Approval No. 2018-237).

### 2.2. Data measurement and collection

Comprehensive health interviews were carried out in person to gather data on various aspects including demographics, such as age, gender, educational level, and income level, lifestyle factors (e.g., smoking, alcohol consumption, and physical activity). As previously described, smoking and alcohol consumption status were assessed through a questionnaire (12). The assessment of physical activity was conducted using the International Physical Activity Questionnaire-Long (IPAQ-L) (21).

Measurements of body weight with a precision of 0.1 kg, height, and waist circumference (WC) with a precision of 0.1 cm were taken. Body mass index (BMI) was calculated by dividing weight in kilograms by the square of height in meters (kg/m^2^). Blood pressure was measured twice while seated, following at least 5 min of rest, using a standardized automatic electronic sphygmomanometer (Omron HBP-9020, Kyoto, Japan). Blood samples were collected after a minimum of 8 h of overnight fasting and sent to the laboratory of local medical institutions for analysis of fasting blood glucose (FBG), triglycerides (TG), total cholesterol (TC), high-density lipoprotein (HDL)-cholesterol (HDL-C), and low-density lipoprotein (LDL)-cholesterol (LDL-C) levels in plasma.

### 2.3. Dietary assessment

Face-to-face interviews were used to assess dietary intake using a Simplified Food Frequency Questionnaire (SFFQ), which was described previously (12). The SFFQ incorporates dietary patterns of Jiarong Tibetan characteristic foods including zanba, a type of coarse cereal, and Tibetan ghee, a dairy product made from milk or goat’s milk. Previous evaluations have been conducted to assess the reliability and validity of this SFFQ (22). Subsequently, intake of each food was estimated by multiplying the frequency of consumption by the standard portion size to obtain a mean daily consumption. The nutrient intake was determined by multiplying the quantity of each consumed food item by its corresponding nutrient content (23).

### 2.4. Statistical analysis

Data were processed using Epidata 3.0 software, and statistical analysis was performed *via* SPSS 19.0 software (IBM, Armonk, NY, USA). Baseline features were described as a mean (standard deviation, *SD*) for continuous variables and the number (proportion) for categorical variables. The categorical variables were compared by the *χ^2^* test. To test the linear trend across BMI groups, the Mantel–Haenszel *χ*^2^ statistic was utilized. Multivariate logistic regression models were used to explore the correlation between diet and biochemical indicators. A two-sided *p* < 0.05 was considered statistically significant.

## 3. Results

### 3.1. Participant characteristics

A total of 476 participants were included in this study. Among them, 41 participants lived at altitudes of 1,500 to 2,399 m, 277 at altitudes of 2,400 to 3,299 m, and 158 at altitudes of 3,300 m or above. Participants’ characteristics are listed in Table 1. Participants aged between 18 and 80 years old, with an average age of 44.33 ± 14.90 years old. There were 263 (55%) and 213 (44%) male and female participants, respectively (The flow chart see Supplementary Figure S2).

**Table 1:** Characteristics of participants at different altitudes.

Variables	Total (*n* = 476)	Altitude (m)		
		1,500 ~ 2,399, *n* = 41	2,400 ~ 3,299, *n* = 277	≥3,300, *n* = 158
Gender (*n*, %)				
Male	263	22 (53.70)	156 (56.30)	85 (53.80)
Female	213	19 (46.30)	121 (43.70)	73 (46.20)
Age (*n*, %)				
18–39	216	9 (22.00)	136 (49.1)	71 (44.90)
40–59	158	18 (43.90)	87 (31.40)	53 (33.50)
60–80	102	14 (34.10)	54 (19.50)	34 (21.50)
Education level (*n*, %)				
Elementary school or lower	207	28 (68.30)	74 (26.70)	105 (66.5)
Middle school	88	6 (14.60)	62 (22.40)	20 (12.70)
High school or above	181	7 (17.10)	141 (25.90)	33 (20.90)
Family income (*n*, %)				
≤2500 RMB per month	92	34 (85.00)	0 (0)	58 (36.70)
2501–4999 RMB per month	96	3 (7.50)	0 (0)	93 (58.9)
≥5,000 RMB per month	287	3 (7.50)	277 (100.00)	7 (4.4)
Smoking (*n*, %)				
Never	268	31 (75.60)	139 (50.20)	268 (56.30)
Yes	208	10 (24.40)	138 (49.80)	60 (38.00)

### 3.2. Dietary characters

Table 2 shows the intake of food groups, nutrients, and physical activity of Jiarong Tibetans in different altitudes. Participants living at higher altitudes tended to consume more red meat and less poultry, aquatic products, vegetables, and fruits. In terms of nutrients, compared with LA and MA groups, participants in the HA group had higher calorie nutrients. For vitamins and minerals, HA group consumed more vitamin A, vitamin C, vitamin E, calcium, and iron (*p* < 0.05).

**Table 2:** The diets and physical activity of Tibetans at different altitudes.

Variables	Altitude (m)			
Food groups (g/d)	1,500 ~ 2,399	2,400 ~ 3,299	≥3,300	*F/χ^2^* value
Cereal	181.20 (45.94)	181.54 (81.45)	122.66 (46.60)	11.57**
Bread and noodle	66.68 (49.11)	65.27 (45.09)	96.99 (58.84)	9.32**
Whole grain (including Zanba)	34.97 (53.87)	75.99 (62.98)	170.84 (139.01)	44.06**
Red meat	55.53 (60.50)	119.97 (53.71)	229.17 (163.76)	41.81**
Poultry	19.79 (20.85)	11.05 (15.33)	8.25 (9.34)	4.55*
Aquatic product	7.74 (10.61)	2.73 (5.00)	1.52 (3.66)	10.22**
Animal organ and processed meat	10.73 (13.37)	17.24 (25.72)	22.94 (12.07)	4.21*
Egg	12.88 (25.46)	17.93 (18.65)	28.45 (33.51)	9.11**
Dairy	19.35 (16.41)	43.47 (28.97)	197.58 (188.21)	69.09**
Vegetable	132.68 (69.99)	109.29 (45.83)	78.51 (100.93)	4.52*
Phycomycete	2.08 (5.29)	2.44 (3.99)	5.53 (8.55)	4.53*
Soy product	14.26 (11.11)	13.30 (10.84)	10.49 (10.43)	2.30
Fruit	24.65 (41.26)	24.97 (26.15)	29.59 (32.46)	3.05
Nut	5.10 (13.07)	5.38 (23.43)	5.83 (9.39)	2.40
Ghee	24.75 (32.62)	37.25 (26.58)	125.88 (90.81)	48.98**

Nutrients	1,500 ~ 2,399	2,400 ~ 3,299	≥3,300	*F/χ^2^* value
Energy (kcal/d)	1751.02 (433.83)	2011.92 (454.86)	3449.69 (843.70)	68.09**
Protein (g/d)	50.51 (34.12)	62.76 (10.81)	91.01 (21.34)	11.31**
Carbohydrate (g/d)	231.16 (88.47)	245.66 (102.32)	337.65 (123.24)	43.12**
Fat (g/d)	74.25 (19.41)	93.51 (20.78)	191.63 (74.36)	62.43**
Alcohol (g/d)	13.79 (11.56)	18.43 (23.24)	27.84 (26.79)	4.53*
Dietary fiber (g/d)	6.63 (2.13)	6.48 (2.34)	5.98 (3.07)	1.88
Vitamin A (ugRE/d) ^a^	197.41 (89.47)	257.05 (124.72)	700.55 (204.83)	116.23**
Vitamin B1 (mg/d)	1.36 (0.43)	1.21 (0.20)	1.46 (0.91)	1.66
Vitamin B2 (mg/d)	0.64 (0.11)	0.70 (0.31)	1.07 (0.62)	1.91
Vitamin C (mg/d)	50.77 (24.70)	56.50 (31.31)	63.74 (27.4)	8.24**
Vitamin E (mg/d)	23.2 (9.31)	24.83 (8.87)	29.91 (10.42)	3.62*
Calcium (mg/d)	320.88 (158.32)	369.39 (173.41)	717.15 (221.34)	86.24**
Iron (mg/d)	14.21 (7.14)	15.38 (8.21)	20.85 (9.38)	4.21*

Physical activity	1,500 ~ 2,399	2,400 ~ 3,299	≥3,300	*F/χ^2^* value
Total activity (MET/week)	572.50 (400.82)	723.91 (738.21)	1208.07 (903.25)	7.62**
Light-intensity activity				
Frequency/week	3.86 (2.21)	4.51 (1.72)	3.61 (2.14)	24.91**
Minutes/time	19.41 (11.43)	26.10 (12.67)	48.44 (42.32)	23.94**
Moderate-intensity activity				
Frequency/week	2.14 (1.89)	1.14 (1.69)	1.96 (1.20)	4.30**
Minutes/time	23.64 (18.66)	10.16 (14.95)	54.27 (67.86)	36.69**
High-intensity activity				
Frequency/week	0.36 (0.73)	0.58 (1.06)	1.09 (1.57)	4.69**
Minutes/time	6.82 (15.16)	16.74 (28.62)	34.9 (38.68)	9.16**

Data are expressed as mean (Standard deviation, SD).

^a^Sum of retinol, β-carotene, α-carotene, and cryptoxanthin.

MET, Metabolic equivalent of energy.

^*^p < 0.05, **p < 0.01.

**Figure 1:**
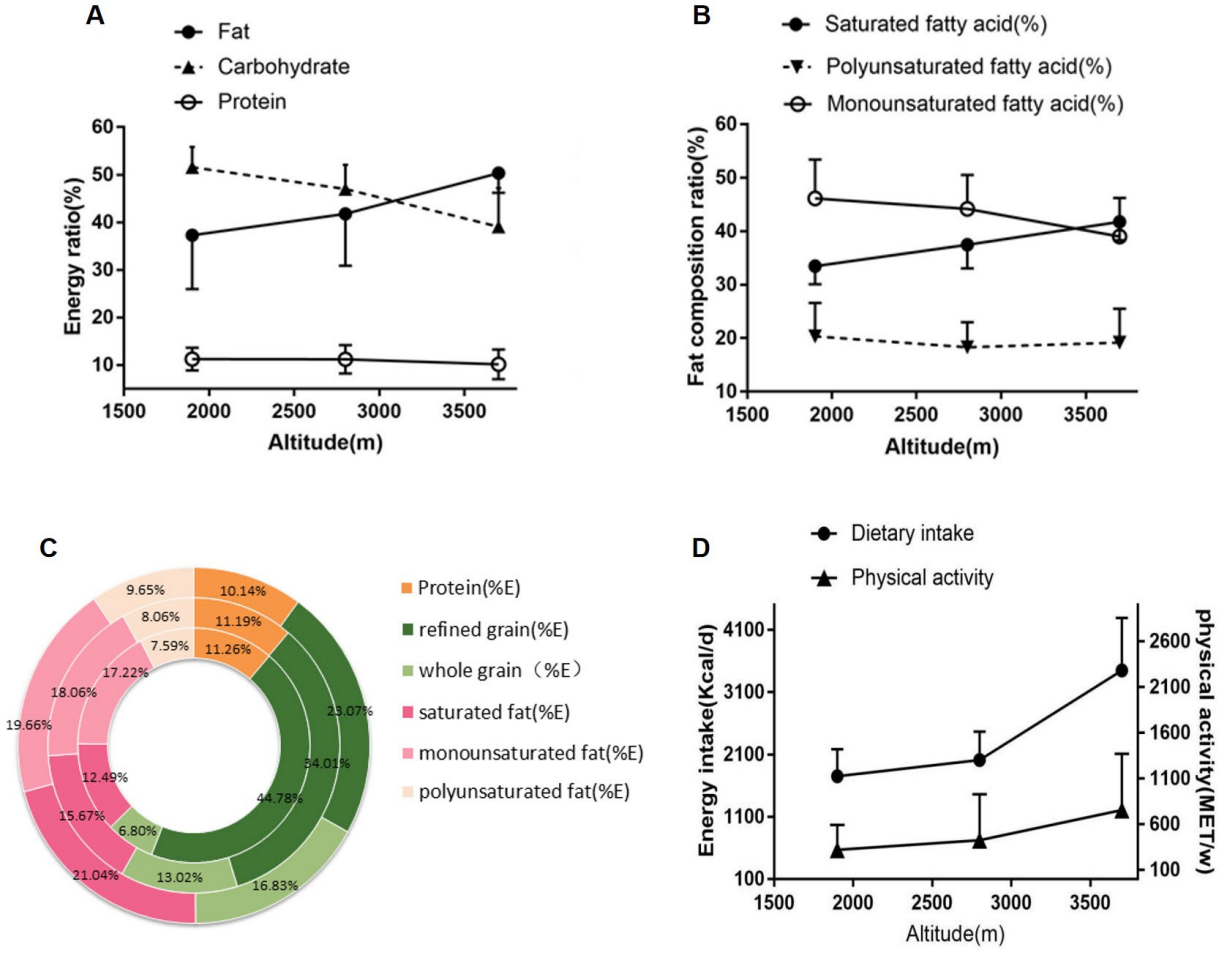
Diet composition and physical activity of Tibetans at different altitudes. **(A)** Energy supply ratio (%) of Tibetans at different altitudes. **(B)** Fat composition ratio (%) of Tibetans at different altitudes. **(C)** Detailed comparison of energy supplement ratio (%) of Tibetans at different altitudes. The inner circle represents the macronutrient energy supply ratio of 1,500 ~ 2,399 m, the middle circle represents that of 2,400 ~ 3,299 m, and the outer circle represents that of 3,300 m or above. %E: The percentage of energy supplement. **(D)** Energy intake and physical activity of Tibetans at different altitudes. MET/w: Metabolic equivalent of energy/week.

Figures 1A,B illustrate the energy supply ratio (%) and fat composition ratio (%) of Tibetans at different altitudes. The dietary energy composition of fat gradually increased with altitude, while the ratio of carbohydrate significantly decreased. There was no significant change in the ratio of protein.

Figure 1C describes the detailed comparison of energy supplement ratio (%) at different altitudes, in which the altitude increased by the circle from inside to outside. With the elevation of altitude, the proportions of protein (E%) and refined grains (E%) decreased, while the proportions of whole grains (E%), saturated fatty acid (E%), monounsaturated fatty acid (E%), and polyunsaturated fatty acid (E%) increased.

### 3.3. Physical activity

Participants’ physical activity at different altitudes is presented in Table 2. Physical activity was divided into light-, medium-, and high-intensity, as assessed by the IPAQ-L (21). The number of times per week and the duration in minutes per session were specified. Both the LA and MA groups were engaged in the light-or moderate-intensity physical activity, whereas the HA group primarily took part in the high-intensity physical activity, and their activity sessions lasted longer. The total amount of activity significantly increased with the elevation of altitude. Figure 1D more clearly shows the variation of energy intake versus physical activity with the elevation of altitude.

### 3.4. Physical and biochemical indicators

Physical and biochemical indicators of Tibetans at different altitudes are presented in Table 3. Systolic blood pressure (SBP) and diastolic blood pressure (DBP) showed statistically significant differences in the altitude partitions. The indicators of BMI, WC, TC, HDL-C, and LDL-C showed an inverted U-shape relationship among the three altitudes, in which the maximum value was at the altitude of 2,400 ~ 3,299 m.

**Table 3:** Physical and biochemical indicators of Tibetans at different altitudes.

Variables	1,500 ~ 2,399 m	2,400 ~ 3,299 m	≥3,300 m	*F* value
BMI (kg/m^2^)	22.52 (1.86)	23.17 (2.51)	23.49 (3.10)	2.25
WC (cm)	83.829 (10.25)	92.17 (11.95)	91.38 (12.26)	4.92**
TG (mmol/L)	1.43 (0.85)	1.44 (0.95)	1.17 (0.92)	4.49*
TC (mmol/L)	4.18 (0.82)	4.73 (0.91)	4.59 (1.08)	6.17**
HDL-C (mmol/L)	1.32 (0.45)	1.28 (0.31)	1.46 (0.40)	11.66**
LDL-C (mmol/L)	2.34 (0.60)	2.61 (0.67)	2.35 (0.86)	7.70**
SBP (mmHg)	134.27 (14.91)	127.36 (11.80)	129.50 (15.41)	5.23**
DBP (mmHg)	73.93 (8.90)	78.38 (9.59)	81.91 (12.06)	11.45**
FBG (mmol/L)	4.90 (0.68)	5.61 (1.49)	5.09 (2.20)	6.50*

BMI, body mass index; WC, waist circumference; TG, Triglycerides; TC, Total cholesterol; LDL-C, low-density lipoprotein cholesterol; HDL-C, high-density lipoprotein cholesterol; SBP, Systolic blood pressure; DBP, Diastolic blood pressure; FBG, fasting blood glucose.

Data are expressed as mean (Standard deviation, SD).

^*^p < 0.05, **p < 0.01.

Multivariate logistic regression models were used to explore the correlation between diet and biochemical indicators. Spearman’s rank correlation coefficient was used to determine descriptive relevance. After adjusting for age, educational level, monthly income, and smoking status, the relationship and statistical differences between dietary intake and related indicators are shown in Table 4. TG and BMI were positively correlated with energy excess and high intake of refined food. However, the LDL-C and TC levels in the HA group were negatively correlated with the percentage of energy supplied by fat. The LDL-C and TC levels were positively correlated with the E% of saturated fatty acids (SFA) and negatively correlated with the E% of unsaturated fatty acids (USFA).

**Table 4:** Association between dietary intake and related indicators.

Variables	Altitude (m)	Spearman’s rank correlation coefficient^#^						
		ΔEnergy	Carbohydrates (%E)	Flour and rice (%E)	Whole grain (%E)	Fat (%E)	SFA(%E)	USFA(%E)
BMI (kg/m^2^)	1,500 ~ 2,399	0.53**	0.20	0.47*	0.08	0.26	0.53*	−0.037
	2,400 ~ 3,299	0.22**	0.065	0.46*	0.15	0.35*	0.14*	0.008
	≥3,300	0.36*	0.15	0.52**	−0.17	−0.28	−0.24	−0.16
WC (cm)	1,500 ~ 2,399	0.38*	0.15	0.56*	−0.31*	0.18	0.18	0.051
	2,400 ~ 3,299	0.20	0.12	0.08	−0.24*	0.13	0.18*	0.046
	≥3,300	0.15	0.06	0.27	−0.15	−0.28	−0.32*	−0.13
TG (mmol/L)	1,500 ~ 2,399	0.59**	−0.021	0.54**	0.19	0.12	0.66**	−0.19
	2,400 ~ 3,299	0.32*	0.022	0.24*	−0.023	0.043	0.15*	−0.01
	≥3,300	0.40**	0.36*	0.30*	−0.17	−0.27	0.41*	−0.26
TC (mmol/L)	1,500 ~ 2,399	0.13	−0.22	0.51*	0.13	0.37*	0.51*	−0.15
	2,400 ~ 3,299	0.035	0.14	0.12	0.094	0.15*	0.082	−0.12
	≥3,300	0.38	−0.17	0.19	−0.24	−0.41**	0.45*	−0.31*
HDL-C (mmol/L)	1,500 ~ 2,399	0.34	−0.24	0.027	0.29	0.12	−0.23	0.013
	2,400 ~ 3,299	0.018	−0.006	0.032	0.13	0.039	−0.056	0.03
	≥3,300	−0.38	0.013	−0.096	0.54*	0.16	0.072	0.22
LDL-C (mmol/L)	1,500 ~ 2,399	0.025	−0.16	0.23	0.21	0.21*	0.16	−0.084
	2,400 ~ 3,299	0.055	−0.17	0.17*	−0.016	0.18*	0.25	−0.17*
	≥3,300	0.51**	−0.075	0.48**	−0.52**	−0.55**	0.49**	−0.48**

# Spearman’s Rank Correlation Coefficient, *p* value were adjusted for age, education level, monthly income and smoking status.

SFA, Saturated fatty acid; USFA, Unsaturated fatty acid.

%E: The percentage of energy supplement.

ΔEnergy (kcal/d) = intake energy (kcal/d) – physical activity consumes (kcal/d).

* Compared with the Spearman’s Rank Correlation Coefficients of the three altitude partitions, *p* < 0.05.

** Compared with the Spearman’s Rank Correlation Coefficients of the three altitude partitions, *p* < 0.01.

## 4. Discussion

The high-altitude adaptation of Tibetans is an exceptional example of natural selection during recent human evolution. People residing at higher altitudes exhibit specific physiological modifications, such as elevated hemoglobin concentrations, augmented lung volume, and decreased hypoxic ventilation response (24–26). Three regions were widely recognized as highland: the Siman Mountains of Ethiopia, the Tibetan Plateau, and the Andean Plateau (27). However, there was no common pattern of adaptations among the populations of these three regions (10, 28). For example, Sherpa and Aymara, who also resided at high altitudes, had different compositions and potential functions of gut microbiota (29). The diverse suitable strategies for each population to cope with their respective extreme environments may have resulted from genetic, geographic, dietary structural, and cultural differences in the hosts (30). Twenty-five Tibetan-specific loci, located in altitude adaptation-related genes such as EPAS1 and ATP6V1E2 on chromosome 2 hypoxia-inducible factor pathway, exhibited significant differences between the Tibetan and plain populations. Moreover, the frequency distribution of alleles in different Tibetan populations showed regional specificity (31). Jiarong Tibetan, a branch of the Tibetan ethnic group, has resided in the Minjiang River basin at an altitude ranging from 500 to 4,000 m. The region is located adjacent to the Tibetan Plateau and the Chengdu Plain. Jiarong Tibetan’s formation can be traced back to the Tang Dynasty when the Tubo people moved eastward, and their genes assimilated with those of the Qiang people (20). The Tibetan-Yi Corridor, situated in a significant location between the northwest and southwest regions of China, has played a crucial role in the migration of various ethnic groups. The unique historical and geographical context has led to Jiarong Tibetans exhibiting distinct mixed characteristics that set them apart from other Tibetan subgroups. The analysis of mutant allele frequency and genetic relationship network diagrams reveals significant differences in the genetic structure of Jiarong Tibetans compared to other Tibetan populations (32). It was found that Jiarong Tibetan had the closest genetic distance to Sichuan Yi people, but was significantly distant from other Tibetan subgroups (33). Advancements in poverty alleviation and medical reform plans of the Chinese government have led to changes in the lifestyle and family income of the Aba area. Numerous ethnic groups inhabit low-altitude areas. Jiarong Tibetans compete with other ethnic groups for job opportunities. However, in the concentrated communities of Jiarong Tibetans living in the middle-altitude Aba area, they hold more jobs in various social and economic activities. Furthermore, the government provides extra subsidies to all residents in the plateau region. As a result, the income of inhabitants in the middle-and high-altitude areas is significantly higher than that of those in low-altitude regions. Notably, while low-altitude regions are suggested for farming and horticulture in several high-altitude mountain areas of the world, high-altitude areas often have abundant pastures that support animal husbandry. In contrast, the Aba area boasts favorable soil conditions and ample sunshine, making it perfect for cultivating fruits, such as alpine apples, plums, seabuckthorn, and snow peer (see Supplementary material 3). Plateau fruit has become one of the primary economic products in the region. Jiarong Tibetans consume relatively more fruits at high altitudes (as evidenced by Table 2), distinguishing their dietary habits from other Tibetan subgroups. Due to their similar genetic background, culture, and lifestyle, Jiarong Tibetans may serve as an excellent population for studying the effects of environmental factors on the physiology.

Altitude had a significant impact on the human energy balance. This was supported by Table 2, which showed a noticeable increase in Jiarong Tibatan’s daily energy intake with altitude, which is consistent with findings from prior research (11). The findings of the present study indicated that the HA group had a significantly higher level of physical activity compared with the control group, as evidenced by the longer duration of all three types of activity intensities (Table 2 and Figure 1D). Moreover, high-intensity activity was more frequent at the high altitude. This could be attributed to the less developed transportation network and challenging terrain in these areas. Although urbanization has led to improvements in several roads, walking or horseback riding remains a common mode of transportation in high-altitude regions, resulting in the increased physical activity. It is well-known that regular exercise reduces the risk of CVD. The present study supported this notion, as moderate physical activity was associated with a 26% reduction in CVD risk, while engagement in high-intensity activities was corresponded to a 42% risk reduction (34). According to a recent study, performing the traditional Tibetan guozhuang dance on a regular basis could enhance blood vessel functionality and cerebral hemodynamics in high altitude environments (35). Despite having a high metabolism and engaging in regular physical activities, Jiarong Tibetans living at high altitudes did not experience a significant increase in BMI, WC, or lipid level in spite of consuming a high energy diet (3449.69 ± 843.70 kcal/d) (Table 3). Notably, multiple studies suggested that weight loss resulting from exposure to high altitude hypoxic environments could be an effective method for reducing body fat in obese individuals (36).

The findings of the present study suggested that diets consumed at high altitudes could be energy-dense, containing high amounts of fat and protein, as revealed by a comparison of calorie composition (11, 37). Consistent with previous studies, the Jiarong Tibetans’ diet in the HA group was found to be rich in fat, providing up to 50% of their energy intake. The consumption of poultry and aquatic products was significantly lower in this group, while the intake of red meat, offal, and processed meat was high. Importantly, although the HA group did not have the highest lipid burden, it had a significantly high content of SFA. Furthermore, an inverted U-shaped change in lipid levels with altitude was observed (Table 4). Remarkably, Ma et al. reported that the prevalence of coronary artery disease (CAD) was lower among Tibetan highlanders compared with plain-living individuals (17). An inverse relationship was reported between altitude and ischemic heart disease (38). The findings of the present study suggested that the high-fat diet adaptation alone might not explain the observations. It was suspected that unique food composition and environmental factors played a crucial role. The higher intensity of physical activity, limited availability of processed food, and efficient metabolism of fat for energy might account for the results at elevated altitudes. Moreover, a significant microbial diversity was found, as indicated by Simpson’s and Shannon’s indices, among the native Tibetans, which would be significantly correlated with carbohydrate metabolism (39). Carbohydrate intake decreased with altitude, while the HA group was more likely to consume whole grains. For instance, Tibetan barley, which could withstand the low temperature and low humidity of the plateau, was a distinctively Tibetan whole grain with high fiber and low fat. Tibetan barley had twice the total soluble phenolic compounds and total antioxidant capacity than barley in the plains, and had the highest β-glucan content (40). A previous study on Tibetan barley concentrated on its ability to significantly regulate blood lipid levels and prevent metabolic syndrome (41). Xia et al. provided an analysis of the contribution of the intestinal microbiota to the hypolipidemic effect of Tibetan barley (42). Another reason was that the intake of female yak milk and ghee was high in plateau areas. The proportions of total polyunsaturated fatty acids (PUFA), total USFA, and the n-3/n-6 FA ratio were high in female yak milk fat and its products (43). Several meta-analyses demonstrated that monounsaturated fatty acids (MUFA) and PUFA reduced both total serum cholesterol and LDL-cholesterol levels, while increased serum HDL-cholesterol level (44, 45). In the inhospitable high-altitude environment, female yak milk and its products were other examples of indigenous diet adapted to living healthily in an extreme environment. In addition, evidence suggested that with every 300 m elevation in altitude, ultraviolet level increased by 10%, leading to the increased vitamin D synthesis at high altitudes (46). It has been reported that vitamin D supplements are associated with a decreased risk of adverse cardiovascular events, such as myocardial infraction, stroke, heart failure, and sudden cardiac death (47). This might be the third reason for the inverted U-shaped change of lipid with altitude.

Several studies have previously concentrated on the influences of genetics, gut microbiota, and plateau environment on the Tibetan population’s ability to metabolize lipids (14, 37, 39). In the present study, the association between Tibetan dietary intake and lipid levels was further analyzed at different altitudes, after adjusting for age, educational level, monthly income, and smoking status (Table 4). When concentrating solely on total energy intake and energy from fat, our results were consistent with previously reported findings (11, 12, 19), where TG and BMI were positively correlated with energy excess and high intake of refined food. However, some notable data could be achieved when comparing groups at different altitudes. Specifically, it was, for the first time, revealed that the LDL-C and TC levels in the HA group were negatively correlated with the percentage of energy supplied by fat. Further analysis indicated that LDL-C and TC levels were positively correlated with the E% of SFA and negatively correlated with the E% of USFA. In addition to the influences of genetic factors, it appears more important to concentrate on the composition of fat intake rather than on the total amount of fat intake among plateau Tibetans. The unique dietary structure consisting of PUFA-rich foods, such as Tibetan barley, whole grains, female yak milk, ghee, and other highland foods may affect the lipid metabolism of plateau Tibetans. Whether and how diet, environment, and genes interact in lipid metabolism in the plateau Tibetan population should be further clarified.

### 4.1. Strengths and limitations

The present study has several strengths. Firstly, to our knowledge, this is the first study to investigate the relationship between lipid levels and Tibetan diets at different altitudes within the same sub-population, thereby reducing potential biases related to ethnic, religious, and traditional cultural factors. Secondly, an inverted U-shaped change was found in lipid level with elevating altitude, despite an increase in fat energy supply ratio, which had not been reported previously. This suggests that diet fat composition may be more important than the amount of fat E% in plateau Tibetans, supplementing previous studies. Further exploration is needed to understand the interaction among diet, environment, and genes in lipid metabolism among the Tibetan population. Finally, our researchers received standardized training to adopt the same measurement methods, generating convincing results.

Nonetheless, this study still has some limitations. Firstly, due to its cross-sectional design, a causal relationship between high-fat diet and lipid level at different altitudes could not be established. Secondly, the self-reported dietary survey might result in reporting bias, although trained interviewers administered it using a standard protocol. Thirdly, the nutritional level was calculated based on the mean value of food composition obtained from our investigation, rather than from actual measurements. The contents of fatty acids and minerals significantly increased with the elevation of altitude in Tibetan Ghee, especially medium-and long-chain UFAs (48, 49). However, the funds obtained for our survey were not enough to support food sampling and experimental analysis. Therefore, there might be a bias between the calculation results and real data, which should be considered during application. Finally, the survey population was relatively small due to differences in religious beliefs, language barriers, and difficult living conditions. Further large-scale research is necessary to obtain a more comprehensive understanding.

## 5. Conclusion

Despite the fact that an increase in fat intake with elevated altitude was found, it was discovered that the Jiarong Tibetans exhibited an inverted U-shaped change in lipid level. The high-fat diet appeared to have varying effects on the lipid level at the high altitude. In addition, it was deemed crucial to concentrate on the composition of fat rather than the total amount of fat expressed as a percentage of energy in the plateau diet. Furthermore, apart from genetic influences, it was suggested that incorporating a diet rich in USFA could potentially counterbalance the risk of lipid metabolism. Nevertheless, the intricate interplay among diet, environment, and genes concerning lipid metabolism in the plateau Tibetan population has still remained obscure. Therefore, further extensive investigations and prospective studies are required to authenticate these findings and provide more comprehensive insights for the future research.

## Data availability statement

The raw data supporting the conclusions of this article will be made available by the authors, without undue reservation.

## Ethics statement

The studies involving human participants were reviewed and approved by the Human Ethics and Research Ethics committees of Sichuan Provincial People’s Hospital (Approval Number: 2018-237), China. The patients/participants provided their written informed consent to participate in this study.

## Author contributions

LT and HY: conceptualization. GJ and QQ: methodology. TX and LT: software and writing-original draft preparation. QQ, GJ, and SP: validation and investigation. QQ and HY: formal analysis. LT and QQ: writing–review and editing. HY: project administration. LT: funding acquisition. All authors contributed to the article and approved the submitted version.

## Funding

This research was funded by Natural Science Foundation of Sichuan Province (grant number: 2022NSFSC0739) and Foundation of Sichuan Provincial People’s Hospital (grant number: 2022RK07).

## Conflict of interest

The authors declare that the research was conducted in the absence of any commercial or financial relationships that could be construed as a potential conflict of interest.

## Publisher’s note

All claims expressed in this article are solely those of the authors and do not necessarily represent those of their affiliated organizations, or those of the publisher, the editors and the reviewers. Any product that may be evaluated in this article, or claim that may be made by its manufacturer, is not guaranteed or endorsed by the publisher.

## Abbreviations

BMI: body mass index
WC: waist circumference
SBP: systolic blood pressure
DBP: diastolic blood pressure
IPAQ-L: International Physical Activity Questionnaire-long
MET: metabolic equivalent of energy
PUFA: polyunsaturated fatty acid
UFA: unsaturated fatty acid
MUFA: monounsaturated fatty acid
*SD*: standard deviation

## References

[1] Center for International Earth Science Information Network, Columbia University: national aggregates of geospatial data: population. Landscape and Climate Estimates, v.2 (PLACE II). (2007). Available at: http://sedac.ciesin.columbia.edu/place

[2] Tremblay JC Ainslie PN. Global and country-level estimates of human population at high altitude. Proc Natl Acad Sci U S A. (2021) 118:e2102463118. doi: 10.1073/pnas.2102463118, PMID: 33903258 PMC8106311

[3] Guo X Long R Kreuzer M Ding L Shang Z Zhang Y. Importance of functional ingredients in yak milk-derived food on health of Tibetan nomads living under high-altitude stress: a review. Crit Rev Food Sci Nutr. (2014) 54:292–302. doi: 10.1080/10408398.2011.584134 24188303

[4] Hirschler V Molinari C Maccallini G Intersimone P Gonzalez CD. San Antonio de los Cobres Study Group Collaborators. Blood pressure levels among indigenous children living at different altitudes. Appl Physiol Nutr Metab. (2019) 44:659–64. doi: 10.1139/apnm-2018-0466 30444642

[5] Hirschler V Gonzalez C Maccallini G Hidalgo M Molinari C San Antonio de los Cobres Study Group Collaborators. Comparison between HDL-C levels in argentine indigenous children living at high altitudes and U.S. children. Diabetes Technol Ther. (2016) 18:233–9. doi: 10.1089/dia.2015.0357, PMID: 27028794

[6] von Wolff M Nakas CT Tobler M Merz TM Hilty MP Veldhuis JD. Adrenal, thyroid and gonadal axes are affected at high altitude. Endocr Connect. (2018) 7:1081–9. doi: 10.1530/EC-18-0242, PMID: 30352395 PMC6198189

[7] Zhang X Zhang Z Ye R Meng Q Chen X. Prevalence of hypertension and its relationship with altitude in highland areas: a systematic review and meta-analysis. Hypertens Res. (2022) 45:1225–39. doi: 10.1038/s41440-022-00955-8 35705740

[8] Koirala S Nakano M Arima H Takeuchi S Ichikawa T Nishimura T. Current health status and its risk factors of the Tsarang villagers living at high altitude in the Mustang district of Nepal. J Physiol Anthropol. (2018) 37:20. doi: 10.1186/s40101-018-0181-y, PMID: 30157969 PMC6114060

[9] Zheng C Chen Z Zhang L Wang X Dong Y Wang J. Metabolic risk factors and left ventricular diastolic function in middle-aged Chinese living in the Tibetan plateau. J Am Heart Assoc. (2019) 8:e010454. doi: 10.1161/JAHA.118.010454, PMID: 30871396 PMC6475067

[10] Li T Shuai P Wang J Wang L. Prevalence, awareness, treatment and control of hypertension among Ngawa Tibetans in China: a cross-sectional study. BMJ Open. (2021) 11:e052207. doi: 10.1136/bmjopen-2021-052207, PMID: 34489294 PMC8422477

[11] Xiao Z Sun X Zhaxi D Zhang F Ji Y Cheng T. Distinct nutrient intake style in inhabitants of ultra-high-altitude areas in north of Tibet, China: a cross-sectional study based on newly developed Tibetan food frequency questionnaires. Front Nutr. (2021) 8:743896. doi: 10.3389/fnut.2021.743896, PMID: 35004798 PMC8733569

[12] Li T Tang X Liu Y Li Y He B. Dietary patterns and metabolic syndrome among urbanized Tibetans: a cross-sectional study. Environ Res. (2021) 200:111354. doi: 10.1016/j.envres.2021.111354, PMID: 34102164

[13] Liu K Yang J Yuan H. Recent progress in research on the gut microbiota and highland adaptation on the Qinghai-Tibet Plateau. J Evol Biol. (2021) 34:1514–30. doi: 10.1111/jeb.13924, PMID: 34473899

[14] Li J Sun L He X Liu J Wang D Han Y. Succession of the gut microbiome in the Tibetan population of Minjiang River Basin. Front Microbiol. (2022) 13:834335. doi: 10.3389/fmicb.2022.834335, PMID: 35479628 PMC9035803

[15] Corbett S Cho JG Ulbricht E Sintchenko V. Migration and descent, adaptations to altitude and tuberculosis in Nepalis and Tibetans. Evol Med Public Health. (2022) 10:189–201. doi: 10.1093/emph/eoac008, PMID: 35528702 PMC9071402

[16] Li L Zhao X. Comparative analyses of fecal microbiota in Tibetan and Chinese Han living at low or high altitude by barcoded 454 pyrosequencing. Sci Rep. (2015) 5:14682. doi: 10.1038/srep14682, PMID: 26443005 PMC4595765

[17] Ma Y Zhu L Ma Z Gao Z Wei Y Shen Y. Distinguishing feature of gut microbiota in Tibetan highland coronary artery disease patients and its link with diet. Sci Rep. (2021) 11:18486. doi: 10.1038/s41598-021-98075-9 34531508 PMC8445913

[18] Liu W Zhang J Wu C Cai S Huang W Chen J. Unique Features of Ethnic Mongolian Gut Microbiome revealed by metagenomic analysis. Sci Rep. (2016) 6:34826. doi: 10.1038/srep34826, PMID: 27708392 PMC5052615

[19] Zhou C Li M Liu L Zhao F Cong W Zhang F. Food consumption and dietary patterns of local adults living on the Tibetan plateau: results from 14 countries along the Yarlung Tsangpo River. Nutrients. (2021) 13:2444. doi: 10.3390/nu13072444, PMID: 34371952 PMC8308694

[20] Dejizhuoga, The origin of Jirarong Tibetan. Tibet Stud. (2004) (2) 51–56.

[21] Shpakou A Naumau IA Krestyaninova TY Znatnova AV Lollini SV Surkov S. Physical activity, life satisfaction, stress perception and coping strategies of university students in belarus during the COVID-19 pandemic. Int J Environ Res Public Health. (2022) 19:8629. doi: 10.3390/ijerph19148629 35886479 PMC9317606

[22] Cheng Z Shuai P Qiao Q Li T. Validity and reliability of a simplified food frequency questionnaire: a cross sectional study among physical health examination adults in southwest region of China. Nutr J. (2020) 19:114. doi: 10.1186/s12937-020-00630-z, PMID: 33023588 PMC7541293

[23] Monteiro LS Hassan BK Estima CCP Souza AM Junior VE Sichieri R. Food consumption according to the days of the week-National Food Survey, 2008–2009. Rev Saude Publica. (2017) 51:93. doi: 10.11606/S1518-8787.2017051006053, PMID: 29020121 PMC5676750

[24] Getu A. Ethiopian native highlander's adaptation to chronic high-altitude hypoxia. Biomed Res Int. (2022) 2022:5749382. doi: 10.1155/2022/5749382, PMID: 35463974 PMC9033342

[25] Salvi P Grillo A Gautier S Montaguti L Brunacci F Severi F. Haemodynamic adaptive mechanisms at high altitude: comparison between European lowlanders and Nepalese highlanders. J Clin Med. (2022) 11:3843. doi: 10.3390/jcm11133843, PMID: 35807128 PMC9267920

[26] Sharma V Varshney R Sethy NK. Human adaptation to high altitude: a review of convergence between genomic and proteomic signatures. Hum Genomics. (2022) 16:21. doi: 10.1186/s40246-022-00395-y, PMID: 35841113 PMC9287971

[27] Valverde G Zhou H Lippold S de Filippo C Tang K López Herráez D. A novel candidate region for genetic adaptation to high altitude in Andean populations. PLoS One. (2015) 10:e0125444. doi: 10.1371/journal.pone.0125444, PMID: 25961286 PMC4427407

[28] Lopez-Pascual A Arévalo J Martínez JA González-Muniesa P. Inverse association between metabolic syndrome and altitude: a cross-sectional study in an adult population of Ecuador. Front Endocrinol (Lausanne). (2018) 9:658. doi: 10.3389/fendo.2018.00658, PMID: 30483215 PMC6240603

[29] Quagliariello A Di Paola M De Fanti S Gnecchi-Ruscone GA Martinez-Priego L Pérez-Villaroya D. Gut microbiota composition in Himalayan and Andean populations and its relationship with diet, lifestyle and adaptation to the high-altitude environment. J Anthropol Sci. (2019) 96:189–208. doi: 10.4436/JASS.97007, PMID: 31782749

[30] Li XN Adnan A Hadi S al-Qahtani WS Alwaili MA Alshaya DS. Genetic characterization of the highlander Tibetan population from Qinghai-Tibet Plateau revealed by X chromosomal STRs. PLoS One. (2022) 17:e0271769. doi: 10.1371/journal.pone.0271769, PMID: 35926061 PMC9352086

[31] Peng Y Yang ZH Zhang H Cui C Qi X Luo X. Genetic variations in Tibetan populations and high-altitude adaptation at the Himalayas. Mol Biol Evol. (2011) 28:1075–81. doi: 10.1093/molbev/msq290, PMID: 21030426

[32] Wang XJ Qian EF Li Y Song ZY Zhao H Xie HX. A genetic sub-structure study of the Tibetan population in Southwest China. Yi Chuan. (2020) 42:565–76. doi: 10.16288/j.yczz.19-330, PMID: 32694115

[33] Wang F Wang M Zhang XH Yu KL Zheng LB Yang YJ. Genetic substructure analysis of three isolated populations in Southwest China. Yi Chuan. (2022) 44:424–31. doi: 10.16288/j.yczz.22-013, PMID: 35729699

[34] Kramer A. An overview of the beneficial effects of exercise on health and performance. Adv Exp Med Biol. (2020) 1228:3–22. doi: 10.1007/978-981-15-1792-1_1 32342447

[35] Li G Zhu A Huang Y Meng J Ji L Xue J. The effect of traditional Tibetan guozhuang dance on vascular health in elderly individuals living at high altitudes. Am J Transl Res. (2020) 12:4550–60. PMID: 32913528 PMC7476110

[36] Pramsohler S Burtscher M Rausch L Netzer NC. Weight loss and fat metabolism during multi-day high-altitude sojourns: a hypothesis based on adipocyte signaling. Life (Basel). (2022) 12:545. doi: 10.3390/life12040545, PMID: 35455035 PMC9026814

[37] Barrera EC Martinez EZ Brunaldi MO Donadi EA Sankarankutty AK Kemp R. Influence of high altitude on the expression of HIF-1 and on the prognosis of Ecuadorian patients with gastric adenocarcinoma. Oncotarget. (2022) 13:1043–53. doi: 10.18632/oncotarget.28275, PMID: 36128327 PMC9477223

[38] Bernabe-Ortiz A Carrillo-Larco RM. Urbanization, altitude and cardiovascular risk. Glob Heart. (2022) 17:42. doi: 10.5334/gh.1130, PMID: 35837362 PMC9231580

[39] Ma Y Ga Q Ge RL Ma S. Correlations between intestinal microbial community and hematological profile in native Tibetans and Han immigrants. Front Microbiol. (2021) 12:615416. doi: 10.3389/fmicb.2021.615416, PMID: 34234749 PMC8257080

[40] Moza J Gujral HS. Starch digestibility and bioactivity of high altitude hulless barley. Food Chem. (2016) 194:561–8. doi: 10.1016/j.foodchem.2015.07.149, PMID: 26471593

[41] Xia X Li G Xing Y Ding Y Ren T Kan J. Antioxidant activity of whole grain highland hull-less barley and its effect on liver protein expression profiles in rats fed with high-fat diets. Eur J Nutr. (2018) 57:2201–8. doi: 10.1007/s00394-017-1494-z, PMID: 28656391

[42] Xia X Li G Ding Y Ren T Zheng J Kan J. Effect of whole grain Qingke (Tibetan *Hordeum vulgare* L. Zangqing 320) on the serum lipid levels and intestinal microbiota of rats under high-fat diet. J Agric Food Chem. (2017) 65:2686–93. doi: 10.1021/acs.jafc.6b05641 28301146

[43] Zhang J Yang M Cai D Hao Y Zhao X Zhu Y. Composition, coagulation characteristics, and cheese making capacity of yak milk. J Dairy Sci. (2020) 103:1276–88. doi: 10.3168/jds.2019-17231, PMID: 31864739

[44] Li N Jia M Deng Q Wang Z Huang F Hou H. Effect of low-ratio n-6/n-3 PUFA on blood lipid level: a meta-analysis. Hormones (Athens). (2021) 20:697–706. doi: 10.1007/s42000-020-00248-0 33123975

[45] Pei J Zhao Y Huang L Zhang X Wu Y. The effect of n-3 polyunsaturated fatty acids on plasma lipids and lipoproteins in patients with chronic renal failure--a meta-analysis of randomized controlled trials. J Ren Nutr. (2012) 22:525–32. doi: 10.1053/j.jrn.2012.04.005 22698988

[46] Mohanna S Baracco R Seclén S. Lipid profile, waist circumference, and body mass index in a high altitude population. High Alt Med Biol. (2006) 7:245–55. doi: 10.1089/ham.2006.7.245, PMID: 16978137

[47] Manson JE Cook NR Lee IM Christen W Bassuk SS Mora S. Vitamin D supplements and prevention of cancer and cardiovascular disease. N Engl J Med. (2019) 380:33–44. doi: 10.1056/NEJMoa1809944, PMID: 30415629 PMC6425757

[48] Jing B Chen W Wang M Mao X Chen J Yu X. Traditional Tibetan ghee: physicochemical characteristics and fatty acid composition. J Oleo Sci. (2019) 68:827–35. doi: 10.5650/jos.ess19031, PMID: 31413239

[49] Zubieta-Calleja GR Zubieta-DeUrioste N. High altitude pulmonary edema, high altitude cerebral edema, and acute mountain sickness: an enhanced opinion from the high Andes-La Paz, Bolivia 3,500 m. Rev Environ Health. (2022) 38:327–38. doi: 10.1515/reveh-2021-0172 35487499

