# Supplementary material for: Lipid levels in the Jiarong Tibetan’s diet at high altitudes: a cross-sectional survey
Source: Front Nutr. 2023 Jun 26;10:1207710. doi: 10.3389/fnut.2023.1207710 (PMC10330741; doi:10.3389/fnut.2023.1207710)

Fruits in Aba Plateau

1. Plateau green apple: Planting on Tibetan Plateau above altitude of 3000 meters.

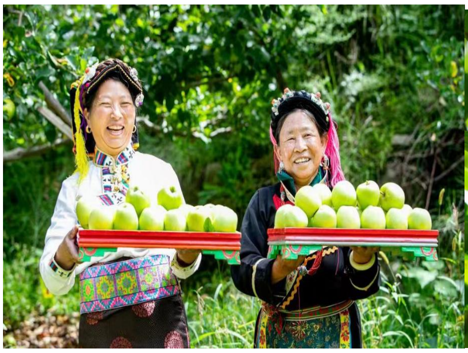
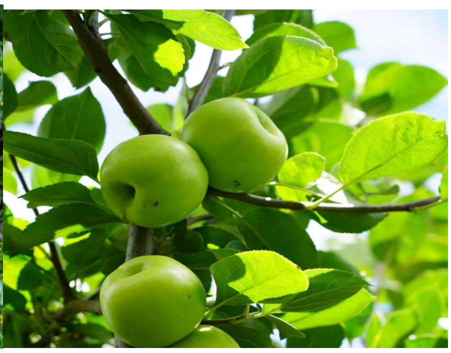

2. Sydney (Snow pear) : located in high mountains and wide valleys at an altitude of 2,000-3,500 meters in Aba area.

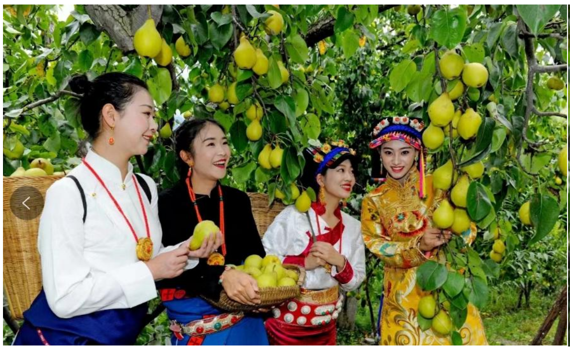
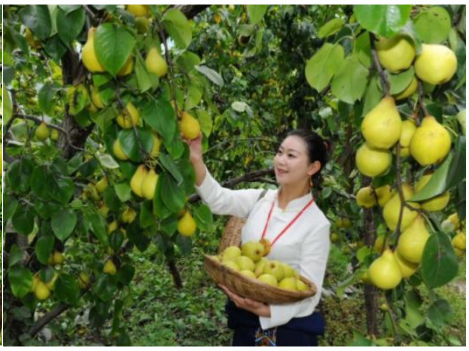

3. Sea buckthorn: grows in temperate regions with an altitude of 800-3600 meters.

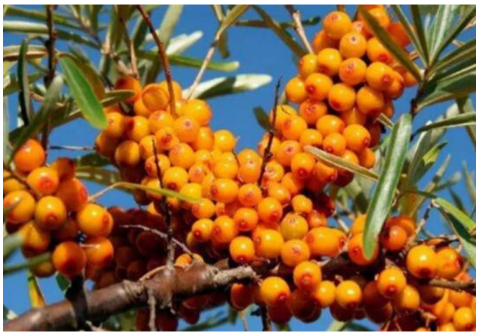
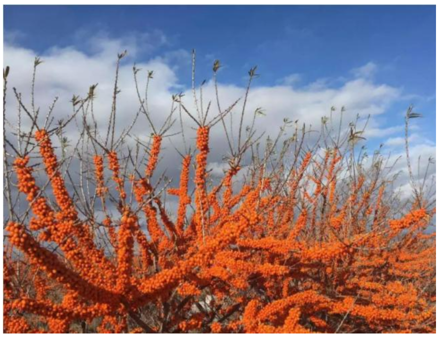

4. Plateau crisp red plum: planted at an altitude of 400-2600 meters.

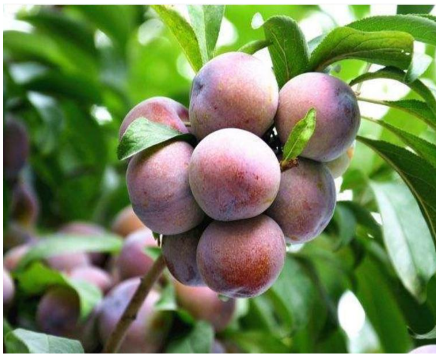
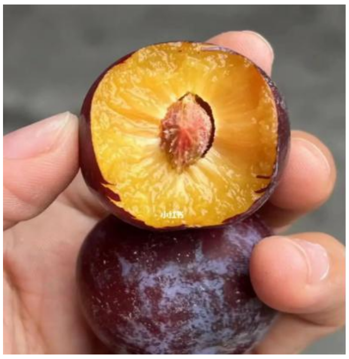

Supplement: Supplementary file 1 [file Data_Sheet_1.pdf]
