# Supplementary figures and images for: Lipid levels in the Jiarong Tibetan’s diet at high altitudes: a cross-sectional survey
Source: Front Nutr. 2023 Jun 26;10:1207710. doi: 10.3389/fnut.2023.1207710 (PMC10330741; doi:10.3389/fnut.2023.1207710)

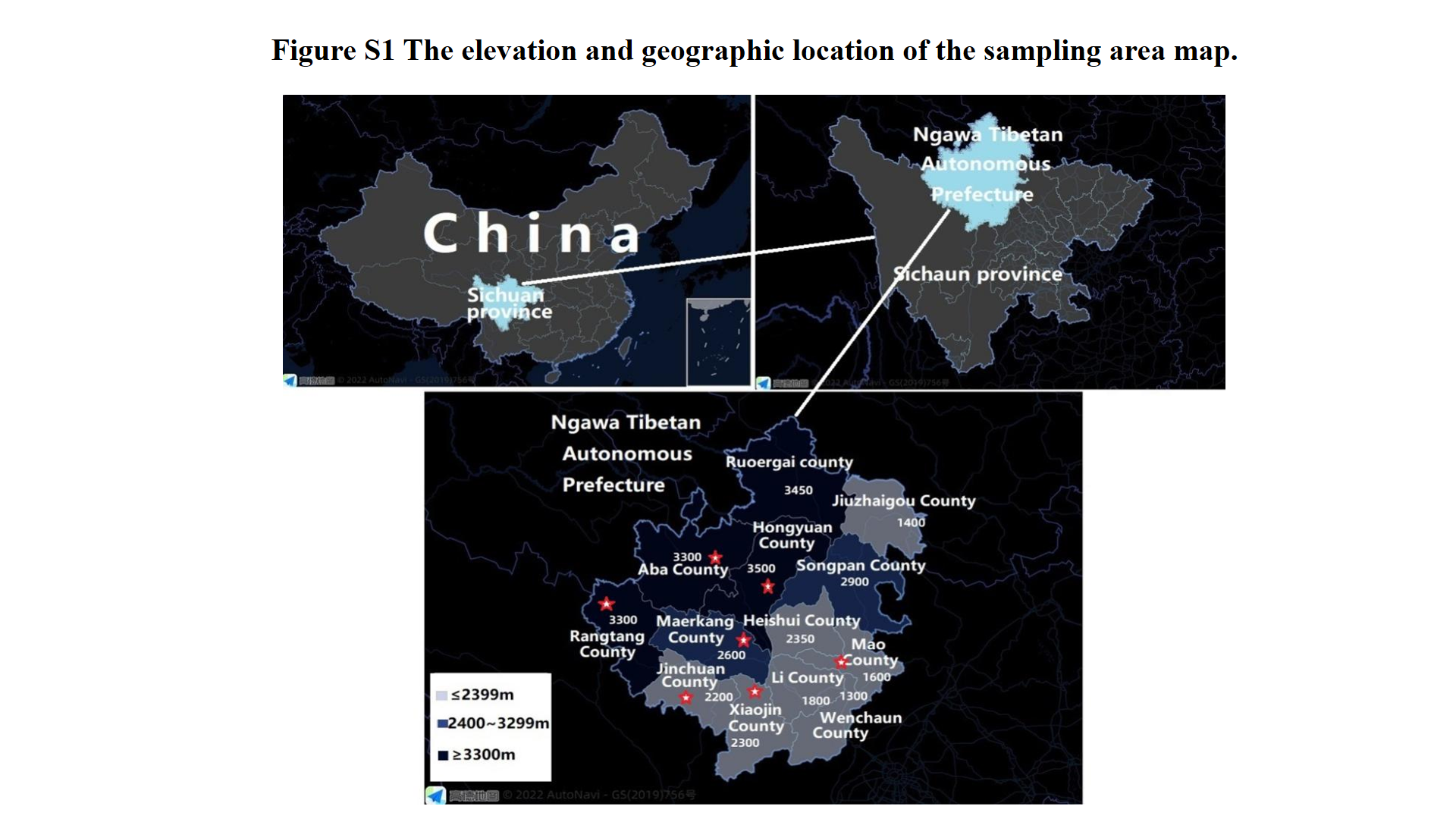

Supplement: Supplementary file 2 [file Image_1.TIF]

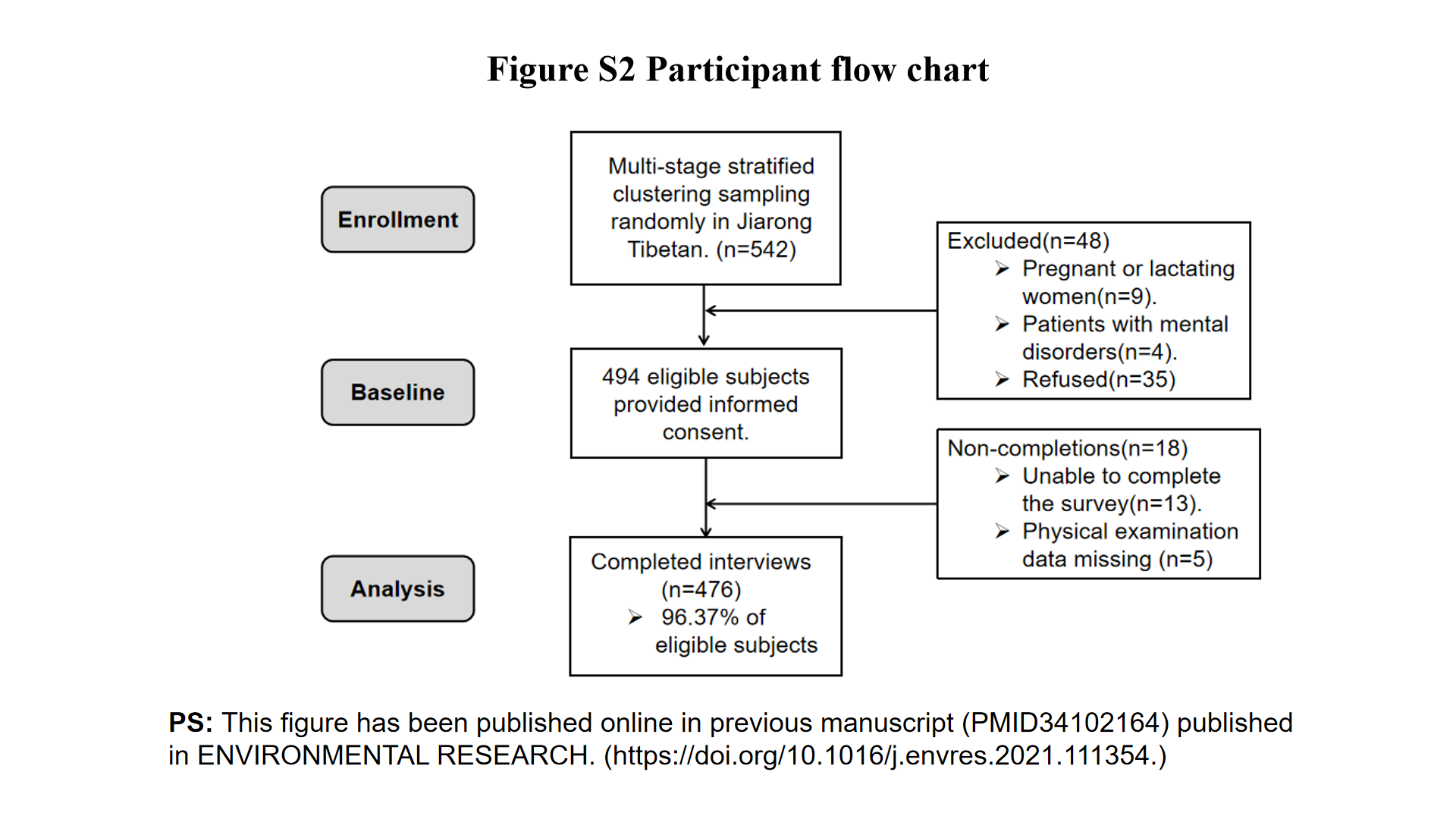

Supplement: Supplementary file 3 [file Image_2.TIF]
